# Organizational culture and climate profiles: relationships with fidelity to three evidence-based practices for autism in elementary schools

**DOI:** 10.1186/s13012-019-0863-9

**Published:** 2019-02-12

**Authors:** Nathaniel J. Williams, Hannah E. Frank, Lindsay Frederick, Rinad S. Beidas, David S. Mandell, Gregory A. Aarons, Philip Green, Jill Locke

**Affiliations:** 1 School of Social Work, Boise State University, 1910 University Drive, Boise, ID 83625 USA; 2 Department of Psychology, Temple University, 1701 N 13th St., Philadelphia, PA 19122 USA; 3 Department of Speech and Hearing Sciences, University of Washington, 1417 NE 42nd St, Seattle, WA 98105 USA; 4 Department of Psychiatry, Perelman School of Medicine, University of Pennsylvania, 3535 Market Street, 3rd floor, Philadelphia, PA 19104 USA; 5 Department of Medical Ethics and Health Policy, Perelman School of Medicine, University of Pennsylvania, Philadelphia, PA USA; 6 Leonard Davis Institute of Health Economics, University of Pennsylvania, Philadelphia, PA USA; 7 Department of Psychiatry, University of California, San Diego, CA USA; 8 Center for Behavioral Health Research, University of Tennessee, Knoxville, TN USA

**Keywords:** Organizational culture, Organizational climate, Implementation, Fidelity, Autism, Schools

## Abstract

**Background:**

Implementation researchers have typically studied organizational culture and climate by testing whether individual dimensions are linked to the implementation of evidence-based practices (EBPs) rather than examining how the overarching social context influences implementation. This approach may limit implementation theory and strategy development to the extent that individual dimensions of culture and climate interact, mutually reinforce or counteract one another, or exhibit non-linear relationships. This study tests whether empirically identifiable culture and climate *profiles* emerge in a sample of organizations and examines how these profiles relate to EBP fidelity and work attitudes that support EBP sustainment, focusing on three EBPs for youth with autism delivered in schools as an example.

**Methods:**

The study included 65 elementary schools in the U.S. that implemented three EBPs—discrete trial training, pivotal response training, and visual schedules—for youth with autism. Organizational culture and climate and work attitudes were assessed using the Organizational Social Context measure at the beginning of the school year. Observations of EBP fidelity occurred mid school-year. We used bias-adjusted stepwise latent profile modeling to (1) identify subpopulations of schools that share similar culture and climate profiles, and (2) test for mean differences across profiles in observed EBP fidelity and teacher and staff work attitudes.

**Results:**

Controlling for region, four profiles best characterized the organizational cultures and climates of schools. Teachers and staff in schools with a *comprehensive* profile (high proficiency culture, positive climate) exhibited higher fidelity to two of three EBPs (*d*’s = .95 to 1.64) and reported superior work attitudes (*d*’s = .71 to 1.93) than teachers and staff in all other schools. Teachers and staff in *supportive* schools (low rigidity culture, positive climate) had better work attitudes, but not better fidelity, than those in schools with *indifferent* (low culture/climate, elevated stress) and *constrained* (high rigidity and resistance, high stress) profiles.

**Conclusions:**

Organizational culture and climate profiles are a strong predictor of EBP fidelity and work attitudes that support EBP sustainment, highlighting the importance of an organization’s overarching social context when developing implementation theory and strategies. Strategies that foster a comprehensive profile may improve EBP implementation.

**Electronic supplementary material:**

The online version of this article (10.1186/s13012-019-0863-9) contains supplementary material, which is available to authorized users.

## Background

Theories and frameworks that explain the implementation of evidence-based practice (EBP) in health care construe an organization’s social context, including its culture and climate, as a general organizational characteristic that affects the organization’s overall functioning and employees’ skillful and effective use of targeted EBPs [1–4]. Consistent with these theories and frameworks, research has linked several individual dimensions of culture and climate to the implementation of EBPs and to factors that support the sustainment of EBPs, such as staff job satisfaction, organizational commitment, and reduced turnover, across a wide range of healthcare settings (see [5] for a review).

This research has led to important advances in identifying targets for implementation strategies; however, this approach to studying culture and climate may underestimate the importance of the total social-psychological context within work environments [6]. One of the goals of implementation research is to understand how the overall social context of a work environment influences the implementation behavior of individuals within that environment [7, 8]. This goal may be compromised when holistic constructs such as culture and climate are broken down into individual dimensions that are measured and analyzed independently [9, 10]. For example, studies that include multiple dimensions of culture or climate in a single linear regression model control for the inter-relationships among these dimensions and implicitly assume that the effects of these variables are additive and independent. This assumption is incorrect if individual dimensions of culture and climate interact, mutually reinforce or counteract one another, or exhibit non-linear relationships such that the overall social context is not equal to the sum of its parts [6]. Recent studies have highlighted the fact that different dimensions of organizational social context interact in complex ways to affect providers’ implementation behaviors [11], underscoring the need for research that takes a nuanced and holistic approach to the study of culture, climate, and EBP implementation. The goal of this study is to examine how overarching configurations or *profiles* of organizational culture and climate relate to fidelity of EBP implementation and to work attitudes that support the sustainment of EBPs.

In a profile approach, investigators empirically identify subpopulations of organizations that share similar response *patterns* across an interrelated set of variables and examine how these subpopulations differ on outcomes of interest [12]. For example, some organizations might have a culture and climate profile that is characterized by lower than average scores on all dimensions, whereas other organizations might have a profile that is high on some dimensions and low on others. These differing profiles reflect the nuanced ways in which individual dimensions of culture and climate move together as they reinforce, attenuate, or otherwise modify each other’s effects. By observing the types of culture and climate profiles that emerge in healthcare organizations and testing differences in how these profiles relate to implementation fidelity and factors that support EBP sustainment (e.g., positive work attitudes and reduced turnover), investigators can gain insight into how an organization’s overarching social context relates to EBP implementation, with implications for developing implementation theory and strategies.

This study focuses on the implementation of three well-established EBPs for youth with autism delivered in public elementary schools in the U.S. Little research has examined the relationships between culture and climate and fidelity to EBPs in schools [13, 14], despite the fact that schools comprise the single largest provider of behavioral health services for youth in the U.S. [15, 16]. Although schools are increasingly expected to implement EBPs [17, 18], researchers have consistently found that EBPs often are not implemented in schools and when they are, fidelity of implementation is typically poor [19–22]. School-based EBPs are especially important for youth with autism, as 52% of these youth receive mental health treatment in school [23]. Identifying factors, such as culture and climate profiles, that are associated with high fidelity to EBPs for youth with autism in schools has strong potential to improve the well-being of this population and to advance implementation theory.

We draw on Glisson and colleagues’ [24] empirically supported theoretical model of organizational culture and climate which was developed specifically to characterize the social contexts of child-serving organizations that deliver behavioral health services. Consistent with the broader literature, this model posits that organizations are characterized by different types of cultures and climates that contribute to differential effectiveness [25–27]. Glisson’s model defines organizational culture as the shared norms and behavioral expectations that guide how work is prioritized and completed in the organization and includes three culture dimensions: proficiency, rigidity, and resistance [24]. Proficiency refers to shared staff perceptions that they are expected to be responsive to client needs and client well-being as their top priority and to maintain competence in up-to-date treatment models. Rigidity describes staff perceptions regarding their autonomy in completing work tasks versus the requirement to closely follow prescribed rules. Resistance describes staff perceptions regarding the extent to which they should actively or passively resist new ways of working and maintain the status quo.

The model defines organizational climate as staffs’ shared perceptions of the impact of the work environment on their personal well-being [28]. The three dimensions of climate in this model are functionality, engagement, and stress [24]. Functionality refers to staff perceptions that they receive support and cooperation from peers and supervisors, clearly understand their role, and have opportunities for growth and advancement. Engagement describes the extent to which staff remain personally involved in their work and accomplish meaningful outcomes. Stress captures shared perceptions of the extent to which role conflict, role overload, and emotional exhaustion interfere with staffs’ ability to do a good job.

Several observational and experimental studies from service settings for youth (e.g., specialty mental health clinics, child welfare agencies) have linked these individual dimensions of culture and climate to superior implementation of EBPs [29–34] and to other outcomes such as service effectiveness and employee work attitudes [35–37]. However, no studies have empirically assessed the overarching culture and climate profiles that may be present in organizations that deliver EBPs nor tested the relationships between these empirically-identified profiles and EBP fidelity. These are important gaps for two reasons. First, implementation theory that describes how multiple dimensions of organizational culture and climate simultaneously influence EBP use has yet to be articulated and thoroughly tested. Many studies assume (either implicitly or explicitly) that culture and climate dimensions have independent and additive effects; these assumptions are embedded within the hypotheses and statistical models. To the extent that these assumptions are incorrect, the models testing them are misspecified and the field’s understanding of social context as a mechanism for supporting EBP implementation and sustainment is at best incomplete and at worst distorted. Investigators may erroneously conclude that organizational social context does not play a role in implementation or that only certain dimensions serve as mechanisms to support implementation when in fact it is a pattern of dimensions (or potentially multiple patterns of dimensions) that together create the necessary and sufficient conditions for successful EBP implementation. Second, these deficits in theory stifle the development of optimally effective and efficient implementation strategies. Drawing on a faulty theory, investigators may develop strategies that are ineffective or they may fail to pursue strategies that might prove more effective or efficient than alternative approaches.

### Study aims

First, we tested the extent to which an interpretable set of organizational culture and climate profiles emerged in a large sample of elementary schools that deliver EBPs to youth with autism. Our primary goal was to empirically identify subpopulations of organizations that shared similar culture and climate profiles. Second, we tested whether these empirically-derived culture and climate profiles were related to two types of outcomes: (1) observed teacher and staff fidelity to three EBPs for youth with autism and (2) teacher and staff job satisfaction and organizational commitment, which comprise work attitudes that are linked to reduced turnover and have been hypothesized as antecedents to improved EBP sustainment [38–40]. Examining both types of outcomes is important because some profiles may support multiple types of EBP-related outcomes (e.g., fidelity, sustainment) whereas other profiles support only a single type of EBP-related outcome.

## Method

### Participants and setting

We conducted an observational study of factors related to fidelity to three EBPs for autism in 65 schools that contained kindergarten to third-grade special education classrooms for youth with autism and that had elected to implement behavioral health EBPs for youth with autism as part of their standard curricula (see [41]). During the last 20 years, a growing number of school districts across the USA have sought to better meet the needs of students with autism by providing training and coaching to their teachers and staff in the use of EBPs based on principles of applied behavior analysis [42]. In partnership with school district officials presiding over districts in the northeastern and northwestern USA that had elected to implement EBPs, our team designed a study to identify factors related to high and low levels of fidelity in these schools in order to generate targets for implementation strategies.

Ninety-two schools were invited to participate in the study because they had a kindergarten-third grade special education classroom with students with autism and planned to provide training and coaching to their staff to implement the target EBPs. Of these 92 schools, 18 declined to participate, 7 had fewer than three staff working in their autism support classroom or provided substantially missing data (i.e., > 30%) on study measures that prevented data aggregation, and 2 were multivariate outliers on study measures, resulting in a final analytic sample of 65 schools (71%), which included 86 kindergarten-through-third grade autism support teachers, 161 classroom staff, and 58 principals serving children with autism. Four schools had three participating autism support classrooms; 13 schools had two participating autism support classrooms, and all other schools had one autism support classroom. Table 1 presents participants’ demographic characteristics.

**Table 1:** Characteristics of study participants

Schools (*N* = 65)	Mean (SD)	Min	Max
Size, # of students	580 (195.86)	290	1225
% free or reduced lunch	77.90 (31.74)	3.6	100
% enrolled in special education services	14.09 (4.87)	6	32
% Black/African American	40.41 (33.27)	< 1	96
% White	25.20 (26.01)	0	79
% Asian	8.36 (10.81)	0	48
% Hispanic/Latino	16.44 (17.09)	1	76
% Native Hawaiian or Other Pacific Islander	.16 (.32)	0	2
% American Indian/Alaskan Native	.24 (.25)	0	1
% Other race	9.18 (3.45)	2	17
School staff			
	Principals *(n = 58)*	Teachers *(n = 86)*	Classroom staff *(n = 161)*
Age in years (M (SD))	47.8 (7.5)	37.5 (11.2)	42.6 (12.5)
Years experience teaching special education (M (SD))	–	8.4 (6.8)	–
	*N* (%)	*N* (%)	*N* (%)
Gender			
Female	37 (63.8)	82 (95.0)	145 (90.1)
Male	21 (36.2)	4 (5.0)	14 (8.7)
Not provided	–	–	2 (1.2)
Race			
White	33 (56.9)	71 (82.6)	86 (53.4)
Black	21 (36.3)	10 (11.6)	59 (36.6)
Asian	2 (3.4)	2 (2.3)	6 (3.7)
American Indian/Alaskan Native	–	2 (2.3)	–
Multiethnic	2 (3.4)	–	3 (1.9)
Not provided	–	1 (1.2)	7 (4.4)
Educational attainment			
High School	–	–	25 (15.5)
Some College	–	–	40 (24.8)
Bachelor’s degree	3 (5.2)	12 (13.9)	48 (29.8)
Graduate/professional degree	53 (91.4)	72 (83.7)	43 (26.7)
Vocational	–	–	4 (2.5)
Other	2 (3.4)	1 (1.2)	1 (0.7)
Not provided	–	1 (1.2)	–

### Evidence-based practices for autism

School districts contracted with a purveyor organization to train all teachers and staff who worked in autism support classrooms in three closely related EBPs which are based on principles of applied behavior analysis (see [41]): discrete trial training (DTT), pivotal response training (PRT), and visual schedules (VS). DTT is a highly structured, one-on-one instructional method that uses massed trials and instructional cues from the teacher/classroom staff to elicit a targeted response from the student with autism [42–44]. PRT is a naturalistic, play-based behavioral intervention that leverages “teachable moments” to train students with autism in generalizable language and play skills (e.g., responsivity to the environment) [45]. VS is a classroom-wide intervention that provides visual cues for students with autism to transition from one activity to another [46]. All three EBPs share a theoretical basis in applied behavior analysis and have strong empirical support [42–46]; consequently, school officials and the purveyor organization presented the EBPs as an integrated set of tools that teachers were expected to use to meet the needs of students with autism related to language, social skills, and pre-academic skills. PRT and DTT are delivered as one-on-one interventions in sessions that typically last approximately 15 min with a student in the classroom [20; 41]. VS is implemented classroom-wide and includes visual prompts that are posted in the classroom or used throughout the day, as well as modifications to how the schedule and transitions are managed [46].

### EBP training and coaching

Training and coaching for the three EBPs occurred via an integrated process. The initial training session occurred prior to the start of the school year and included a two-day interactive learning session as well as distribution of written materials including a manual detailing all three interventions and requisite materials. The initial two-day training focused on the theory and rationale for the interventions, their evidence base, steps in using them in the classroom, and strategies for implementing the interventions via a team approach. Teaching strategies included didactics, video examples, in vivo modeling, role-play, discussion, and “question and answer” sessions focused on application to participants’ classrooms. Teachers were given time to create materials for the visual schedules intervention for use in their classrooms (e.g., individual and classroom schedules). In addition, teachers and staff were provided with lesson plans, toys, flash cards, and data sheets necessary to implement DTT and PRT and they practiced using these materials during the training.

Following the initial training, teachers and staff received monthly coaching in their classrooms related to all three EBPs. Coaching occurred in vivo during class sessions once per month and each session lasted approximately 2 h. Coaches observed teachers’ use of all three EBPs and provided didactic training, modeling, and feedback as well as support with strategies for managing behaviors so that the EBPs could be used to address target behaviors. Additional file 1 presents a Standards for Reporting Implementation Studies (StaRI) checklist with details regarding the implementation strategy used by the schools [47].

### Procedure

The University of Pennsylvania and the University of Washington institutional review boards and the school districts provided ethics approval for the study. Recruitment for the study began by meeting with officials in school districts that had decided to implement EBPs for youth with autism to describe the study, acquire their permission, and obtain a list of elementary schools with self-contained classrooms for children with autism where EBP training would be provided. Next, we contacted the principal at each prospective school to set up an initial meeting and obtain their consent for the school to participate in the study. Following approval by the principal, we met with teachers and school staff who worked in the school’s autism support classroom to obtain their consent to participate in the study. Even if principals agreed to their schools’ participation, teachers and staff could decline survey completion. Training in the EBPs was required by the school districts and was provided to all autism support teachers and staff regardless of their participation in the study and completion of study measures. No modifications to the training or coaching were made as part of the study.

Following recruitment of participants, the study included two phases. First, participants at each school (i.e., principals, autism support teachers, and classroom staff) completed study measures (i.e., organizational culture, climate, and work attitudes) on-site at the beginning of the school year following the EBP training (November–December 2015). Survey completion took approximately 45–60 min and participants were compensated $50 USD for their time. Second, beginning one month after administration of the study measures (i.e., January 2016), a member of the research team visited each school and conducted two classroom observations approximately two months apart to assess fidelity to each EBP in the school’s autism support classrooms. Observations were scheduled during periods of the school day when teachers/staff informed the research team they would be using all three of the EBPs. Observations were conducted in the middle of the school year (January to April) to ensure that teachers and staff had time to learn and implement the EBPs. Data collection was avoided during the beginning (prior to survey administration) and end of the school year when EBP implementation slows.

### Measures

#### Organizational culture and climate

Organizational culture and climate were measured using the Organizational Social Context (OSC) measure, a 105-item instrument that assesses organizational culture, climate, and work attitudes in child-serving settings [24]. Scores on the OSC have demonstrated reliability and validity in several studies [30, 33, 37, 48]. In partnership with the OSC developers, the research team made minor adaptations to the wording of items (e.g., referring to “students” instead of “clients” and “teachers and classroom staff” instead of “members of my organizational unit”) to ensure its use was appropriate among teachers and staff in the school context [41]. Items ask respondents to indicate the extent to which each statement characterizes their workplace on a scale from 1 (*Never*) to 5 (*Always*). Statements refer to shared norms and expectations within the organization (i.e., culture) and the impact of the work environment on the individual’s personal well-being (i.e., climate). Administration takes approximately 20 min.

The OSC provides scores on six subscales reflecting the dimensions of culture (i.e., proficiency, rigidity, resistance) and climate (i.e., engagement, functionality, stress) described above. Subscale scores are generated by aggregating (i.e., averaging) participants’ individual responses to the school-level after evidence of within-group agreement and between-group variance has been provided to support the construct validity of the aggregated, school-level scores (see [49] for details). Aggregation is based on the underlying theory of culture and climate, which posits that these constructs are organization-level variables, not characteristics of individuals [50]. Evidence of staff inter-rater agreement on each OSC scale (i.e., proficiency, rigidity, resistance, engagement, functionality, stress) was provided by the *r*_wg(j)_ statistic [51, 52] where values >  .7 are considered acceptable [53]. In this sample, inter-rater agreement was excellent on all six subscales (mean *r*_wg(j)_ = .92, range = .88 to .97), indicating respondents exhibited high levels of agreement in their perceptions of culture and climate.

The OSC also measures individual-level work attitudes with two scales assessing the individual’s *job satisfaction* and *organizational commitment* [54]. Job satisfaction represents a positive appraisal of one’s job tasks and work assignments, whereas organizational commitment represents a willingness to exert considerable effort on behalf of the organization and a strong desire to remain a member of the organization [55]. In prior studies, these scales have demonstrated excellent reliabilities and evidence of validity [24, 54]. We aggregated individual job satisfaction and organizational commitment scores to the school level to reflect the mean level of these constructs in the schools.

#### EBP Fidelity

Consistent with best practices [19, 20, 56], a research assistant rated DTT, PRT, and VS fidelity via direct observation in each classroom using an EBP-specific fidelity checklist. The checklists were developed in previous studies examining fidelity to these practices in a classroom setting; in prior research, scores on the checklists demonstrated convergent validity with (1) improvement in youth outcomes and (2) self-reported teacher fidelity (see [19, 20, 56]). The fidelity checklists included 11 items for DTT (*α* = .97), 17 items for PRT (*α* = .97), and 10 items for VS (*α* = .93). Items on each checklist assessed the extent to which teachers and staff implemented the core components of the EBP during a 10–15-min observation session using a Likert scale from “0” (does not implement) to “4” (highly accurate implementation). Because teachers and staff within the schools worked as integrated teams to meet the needs of all students within their classrooms, fidelity assessments did not focus on specific teachers or staff per se, but rather on the fidelity with which EBPs were implemented by team members observed during the observation period. Observations of each EBP were limited to 15 min, as this was the allotted time for teachers and staff to work one-on-one with each student with autism to ensure all students in the classroom had the opportunity for one-on-one intervention. VS implementation generally occurred throughout the observation period when students with autism transitioned from one activity to another. For each practice, the items were averaged to calculate a total score for the observation period. Consistent with our conceptualization of teachers and staff as teams that meet the needs of a target student population, fidelity scores for each EBP were averaged across the two observations to yield an average score for each of the three EBPs in each classroom. In schools with more than one autism support classroom, fidelity scores were subsequently aggregated to the school level for use as a school-level outcome variable. Research assistants were trained to 90% reliability on each fidelity measure through didactic instruction and coding of training videos prior to conducting field observations [57].

### Data analysis

We used bias-adjusted stepwise latent profile modeling to identify subpopulations of schools that shared similar culture and climate profiles (conditioned on region) and test for mean differences across profiles on observed EBP fidelity and teacher and staff work attitudes that support EBP sustainment [58–61]. The first step identified subpopulations or classes of schools that shared similar organizational culture and climate profiles based on their organization-level scores on the six subscales of the OSC (i.e., proficiency, rigidity, resistance, engagement, functionality, stress). Scores on each dimension of culture and climate were standardized prior to analysis and all analyses included region (i.e., northwestern vs. northeastern USA) as a covariate to control for differences in resource constraints, policy environments, and student populations across regions. Consistent with best practices, the best fitting model was selected on the basis of: model fit criteria (i.e., lower values on the sample size-adjusted Bayesian information criterion indicate better fit; [62, 63]); entropy (i.e., values closer to 1 indicate greater separation between classes; [64]); sufficiently populated classes (i.e., no less than 5% of the sample in a given class); probabilities of correct classification (i.e., values above .8 are typically considered adequate [65]); and, interpretability of classes based on alignment with previous research and theoretical considerations [65, 66]. In the analysis, the categorical latent variable represents a set of homogeneous subpopulations of schools that share similar organizational culture and climate profiles on the OSC, conditioned on region [61].

The second step tested mean differences across classes on the distal outcomes of EBP fidelity and staff work attitudes using a weighted ANOVA which accounts for the probabilistic uncertainty of classification, as described by Bolck et al. [59], and recommended by methodologists (i.e., the Bolck–Croon–Hagenaars (BCH) approach [60]). This procedure incorporates robust standard errors and uses a Wald test to provide an omnibus test and pairwise comparisons of the equality of means on the distal outcomes across the latent classes. Simulation research indicates the BCH approach yields unbiased estimates and robust tests of significance even when statistical assumptions are violated [58–60, 67]. All analyses were implemented in Mplus, Version 8 via the TYPE = MIXTURE analysis command with the BCH specifier in the AUXILIARY variable command [68]. Effect sizes were calculated using Cohen’s *d* which expresses the distance between means in standard deviation units of the dependent variable [69].

## Results

### School culture and climate profiles

**Fig. 1:**
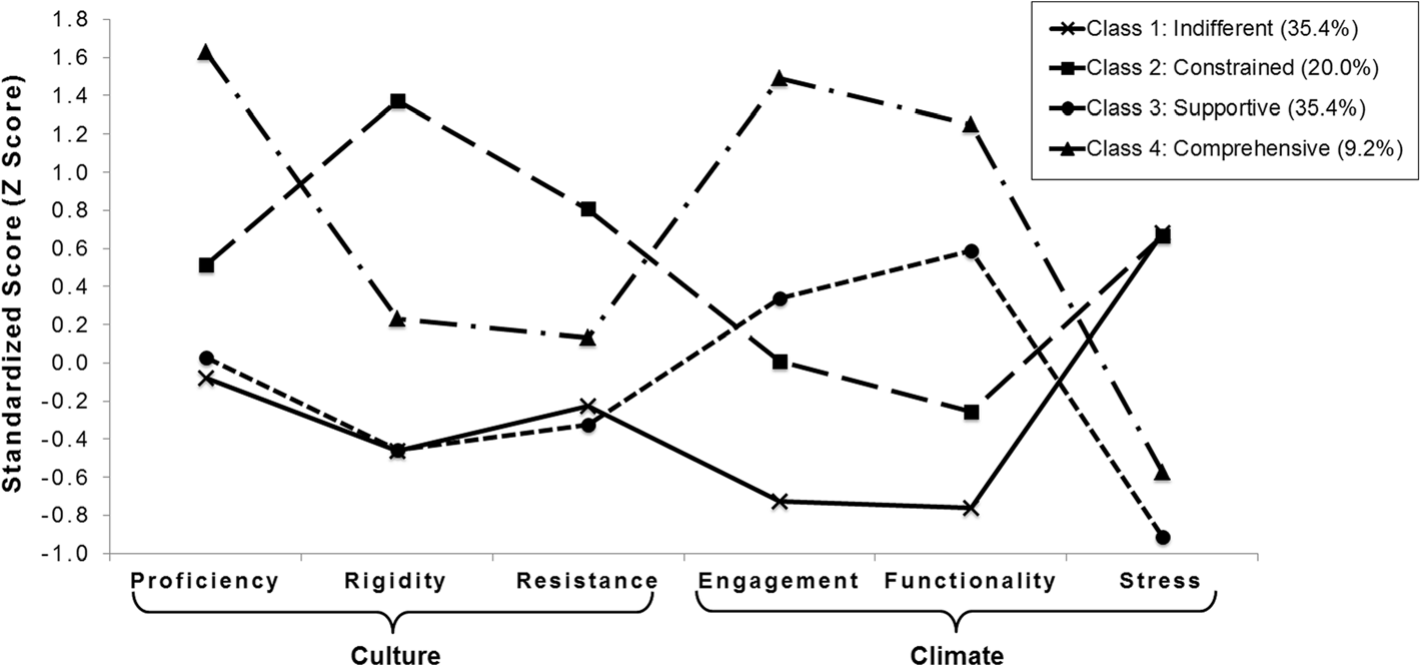
Organizational culture and climate profiles in schools. Note: Organizational culture and climate profiles are based on bias-adjusted stepwise latent profile analysis incorporating the six Organizational Social Context subscale scores and controlling for region. *N* = 65 schools

Results of the latent profile analysis (LPA) indicated a four-class solution best characterized the culture and climate profiles of the schools in this sample, as indicated by model selection criteria (i.e., lowest sample size-adjusted Bayesian information criterion (SSA-BIC) value), good separation between classes (entropy = .87), high average latent classification probabilities (range = .86 to .99), adequate populations in each class (i.e., no classes with < 5% of the sample), and theoretically interpretable culture and climate profiles. Figure 1 shows the culture and climate profiles of the four classes derived from the analysis.

The first profile included 35% of schools (*n* = 23) and was labeled *indifferent*. This profile exhibited low scores on every dimension of culture and climate except stress, which was significantly higher than average (*z* = .68, SE = .22, *p* = .002) and .91 standard deviations higher than any other dimension of culture or climate in the profile. Levels of proficient culture (*z* = − .77, *SE* = .17, *p* < .001), engaged climate (*z* = − .73, SE = .23, *p* = .001), and functional climate (*z* = −.76, SE = .16, *p* = .000) were lower in this profile than in any other profile, suggesting teachers and staff in these schools do not experience norms and expectations to improve student well-being or remain competent in up-to-date practices, nor do they experience the cooperation and support they need from colleagues to perform their jobs. Levels of rigid culture also were significantly lower than average in this profile (*z* = − .46, SE = .16, *p* = .004), indicating teachers and staff perceive minimal oversight or direction from leadership and wide latitude to complete their job tasks.

The second profile included 20% of schools (*n* = 13) and was labeled *constrained.* Schools with a constrained profile were characterized by the highest levels of rigid culture (*z* = 1.37, SE = .25, *p* < .001) and resistant culture (*z* = .81, SE = .28, *p* = .004) observed in the sample, exceeding all other profiles by > 1 and .67 standard deviations, respectively. These scores also exceeded the level of proficient culture in this profile (*z* = .52, SE = .25, *p* = .042). Teachers and staff in these schools report minimal levels of autonomy and strong normative pressure and expectations to reject new ways of working. The most elevated dimension of climate was stress, which was significantly higher than average (*z* = .67, SE = .17, *p* < .001).

The third profile included 35% of schools (*n* = 23) and was labeled *supportive*. These schools had average scores on proficient culture (*z* = .03, SE = .17, *p* = .858) and resistance (*B* = − .33, SE = .20, *p* = .102) and significantly lower than average scores on rigidity (*z* = −.46, SE = .14, *p* = .001), suggesting norms and expectations that support teacher autonomy but are not above average with regard to maintaining competence or adopting new innovations. The most prominent feature of this profile was the pattern of climate scores in which stress was nearly a standard deviation below average (*z* = − .91, SE = .17, *p* < .001) and both functionality (*z* = .59, SE = .21, *p* = .006) and engagement (*z* = .34, SE = .19, *p* = .075) were more than a standard deviation higher than stress. Teachers and staff in these schools share perceptions that their work environment supports their personal well-being and provides relatively high levels of autonomy even as expectations for maintaining competence and incorporating innovations into practice do not exceed average.

The fourth culture and climate profile included 9% of schools (*n* = 6) and was labeled *comprehensive*. The level of proficient culture in this profile (*z* = 1.63, SE = .20, *p* < .001) exceeded any other profile’s level of proficient culture by > 1 standard deviation. This was accompanied by the highest levels of engaged climate (*z* = 1.49, SE = .18, *p* < .001) and functional climate (*z* = 1.25, SE = .46, *p* = .006) observed in the sample. In addition, these schools were characterized by average levels of rigid culture (*z* = .23, SE = .33, *p* = .706) and resistant culture (*z* = .13, SE = .42, *p* = .754) and a slightly lower than average level of stress (*z* = − .58, SE = .44, *p* = .193), reflecting staff perceptions of typical expectations related to the hierarchy of authority (i.e., rigidity) and approach to innovations (i.e., resistance) in a school setting. Teachers and staff in this profile report that they are expected to focus on student well-being and maintain competence in up-to-date practices and they perceive the support and cooperation they need from their colleagues and supervisors to do a good job.

### Relationships between school culture and climate profiles and EBP Fidelity

Table 2 presents the results of the bias-corrected omnibus tests and pairwise comparisons examining mean differences in EBP fidelity across the four culture and climate profiles on each EBP. Teachers and staff in comprehensive schools exhibited significantly higher fidelity to DTT than teachers and staff in supportive (*d* = 1.01, *p* = .010), indifferent (*d* = .95, *p* = .009), and constrained schools (*d* = 1.64, *p* < .001); however, there were no differences in DTT fidelity among teachers and staff in supportive, indifferent, and constrained schools. Similarly, teachers and staff in comprehensive schools exhibited higher fidelity to PRT than teachers and staff in supportive (*d* = 1.21, *p* = .001), indifferent (*d* = 1.21, *p* < .001), and constrained schools (*d* = 1.42, *p* = .001), even as the level of fidelity to PRT in these three school profiles did not differ from each other. There were no significant differences on fidelity to VS across the culture and climate profiles (*p* = .728).

**Table 2:** Differences in fidelity to evidence-based practices and teacher work attitudes by culture and climate profiles

	School culture and climate profiles					
	(Class 1) Indifferent (35%) *n* = 23	(Class 2) Constrained (20%) *n* = 13	(Class 3) Supportive (35%) *n* = 23	(Class 4) Comprehensive (9%) *n* = 6	Omnibus Wald test (*df* = 3)	Significant pairwise comparisons
Criterion variables	*M* (*SE*)	*M* (*SE*)	*M* (*SE*)	*M* (*SE*)		
Fidelity to discrete trial training	2.34 (.22)	1.53 (.37)	2.27 (.25)	3.44 (.36)	13.58**	1 vs. 4** 2 vs. 4*** 3 vs. 4*
Fidelity to pivotal response training	2.05 (.19)	1.83 (.35)	2.05 (.21)	3.27 (.26)	17.96***	1 vs. 4*** 2 vs. 4** 3 vs. 4**
Fidelity to visual schedules	1.43 (.23)	1.62 (.25)	1.80 (.23)	1.73 (.38)	1.30	
Job satisfaction	46.90 (1.33)	44.89 (1.13)	52.50 (1.21)	56.96 (1.60)	48.85***	1 vs. 4*** 2 vs. 4*** 3 vs. 4* 1 vs. 3** 2 vs. 3***
Organizational commitment	44.23 (1.32)	45.66 (1.55)	51.38 (1.50)	56.41 (2.07)	32.62***	1 vs. 4*** 2 vs. 4*** 1 vs. 3** 2 vs. 3*

Note: Wald tests examining the equality of means across latent classes were conducted using bias-corrected stepwise latent profile modeling with a weighted ANOVA as described by Bolck et al. [63]

*p < .05

**p < .01

***p < .001

### Relationships between school culture and climate profiles and collective teacher and staff work attitudes

Results of the bias-corrected omnibus tests and pairwise comparisons examining mean differences on teacher job satisfaction and organizational commitment across the four culture and climate profiles are shown in Table 2. Teachers and staff in schools with comprehensive profiles reported significantly higher job satisfaction than teachers and staff in supportive (*d* = .71, *p* = .033), indifferent (*d* = 1.61, *p* < .001), and constrained (*d* = 1.93, *p* < .001) schools. In addition, teachers and staff in supportive schools reported significantly higher job satisfaction than teachers and staff in indifferent (*d* = .90, *p* = .003) and constrained schools (*d* = 1.23, *p* < .001).

A similar but not identical pattern emerged for organizational commitment. Teachers and staff in comprehensive and supportive schools reported significantly higher levels of organizational commitment than teachers and staff in constrained and indifferent schools even though the levels of organizational commitment did not differ between the top two profiles (comprehensive vs. supportive, *p* = .058) or the bottom two profiles (constrained vs. indifferent, *p* = .493).

### Post hoc analyses

The analyses presented above confirmed that different culture and climate profiles in schools are related to different levels of EBP fidelity and teacher and staff work attitudes, controlling for differences in resources, policies, and populations that characterize the two regions in this study. They do not, however, establish the factors that serve as antecedents to culture and climate profiles or clarify whether some third variable, such as financial strain, relates to both culture and climate profiles and EBP fidelity. While full examination of this issue is beyond the scope of this study, we partially addressed this by testing whether within-region variation in school size and the percent of students who received free or reduced lunch differed across culture and climate profiles. We conceptualized these variables as indicators of financial strain on schools and tested them using the bias-corrected BCH procedure described above.

Results of these analyses are presented in Table 3. Neither variable was supported as an antecedent to, or potential confound of, the relationship between culture and climate profiles and EBP fidelity or work attitudes. Schools with comprehensive profiles did not differ from any other profile on size (measured as the total number of students); although schools with indifferent profiles were significantly smaller than supportive schools (*p* = .041). Schools with comprehensive (96.15%) and constrained (96.88%) profiles did not differ from each other on the percentage of students who received free or reduced lunch (*p* = .921); however, these schools had significantly *higher* percentages of students receiving lunch assistance than schools with indifferent (69.87%) and supportive (69.58%) profiles. These analyses indicate that the positive fidelity outcomes observed in schools with comprehensive profiles are not related to school size or the percent of students receiving free or reduced lunch.

**Table 3:** Differences in school characteristics by school culture and climate profiles

	School culture and climate profiles					
	(Class 1) Indifferent (35%) *n* = 23	(Class 2) Constrained (20%) *n* = 13	(Class 3) Supportive (35%) *n* = 23	(Class 4) Comprehensive (9%) *n* = 6	Omnibus chi-square test (*df* = 3)	Significant pairwise comparisons
School characteristic	M (SE)	M (SE)	M (SE)	M (SE)		
School size (# of students)	497.69 (27.43)	655.44 (75.32)	609.70 (45.17)	592.24 (45.66)	7.96*	1 vs. 3*
Percent students with free/reduced lunch	69.87 (8.42)	96.88 (2.29)	69.58 (6.96)	96.15 (6.97)	22.80***	1 vs. 2** 1 vs. 4* 2 vs. 3*** 3 vs. 4*

Note: Wald tests examining the equality of means across latent classes were conducted using bias-corrected stepwise latent profile modeling with a weighted ANOVA as described by Bolck et al. [63]

*p < .05

**p < .01

***p < .001

## Discussion

This study extends implementation theory and research by using a profile approach to understand how configurations of organizational culture and climate dimensions relate to staff fidelity to three EBPs for youth with autism in public elementary schools [1–3]. Whereas prior studies have investigated the relative importance of individual dimensions of culture and climate for EBP implementation (e.g., [29, 70]), this study sought to understand how the overarching social context of culture and climate relates to EBP implementation outcomes [6, 12]. Results confirm that schools exhibited distinguishable organizational culture and climate profiles that predicted large differences in observed teacher and staff fidelity to EBPs as well as variation in teacher and staff work attitudes that support EBP sustainment. These findings extend prior research by showing that service delivery organizations exhibit distinctive culture and climate profiles—which reflect the unique ways that individual dimensions of culture and climate interact, reinforce, and counteract one another—and that these profiles are strongly related to EBP fidelity and factors that support EBP sustainment.

Results from this study have important theoretical implications and highlight areas for future research. First, no single dimension of culture or climate predicted EBP fidelity on its own nor did the linear combination of all six dimensions predict fidelity in an OLS regression model (see Additional file 2 for a bivariate correlation matrix and regression results). Instead, high fidelity was observed only when a comprehensive configuration of highly proficient culture, average levels of rigidity and resistance, and a highly positive organizational climate were all present. This suggests that dimensions of organizational culture and climate interact with, reinforce, and counteract one another in complex, non-linear ways as they relate to EBP implementation such that the overall gestalt of the social context may be more important than the level of a single dimension [11]. Research examining organizational social context profiles in other industries (e.g., banking, food distribution) suggests that the comprehensive and supportive profiles observed in this study may generalize to other types of organizations and may reflect the organization’s balance between a focus on strategic outcomes (e.g., fidelity) and employee well-being (e.g., climate) [6]. Clearly, there is a need for theory development and testing to better understand the types of culture and climate profiles that emerge in different types of organizations that deliver healthcare services (e.g., schools, hospitals, outpatient clinics), the antecedents to these different profiles, and how these profiles relate to implementation outcomes. Ostroff and Schulte [71] provide a review of methods for studying organizational culture and climate profiles, including the use of qualitative comparative analysis and other fuzzy set methods which focus on identifying the necessary and sufficient conditions for achieving defined outcomes [72].

Second, there is evidence that different profile patterns support different types of outcomes. Supportive profiles were related to superior teacher and staff work attitudes, which is consistent with the support these workplaces provide for staff well-being accompanied by the relatively high levels of staff autonomy. However, supportive profiles were not associated with superior staff fidelity to EBPs, possibly because of the lack of proficient cultural expectations to direct staff toward EBPs as an important part of effective service delivery (i.e., competence in up-to-date treatment practices). In contrast, teachers and staff working in schools with comprehensive profiles, which engendered both highly proficient cultures (along with average rigidity and resistance) *and* highly positive climates, had superior EBP fidelity *and* employee work attitudes. This is consistent with other studies showing an interaction between general organizational climate and more focused dimensions of organizational social context that support targeted behaviors [6, 11, 73]. Based on these results, we can speculate that different types of culture and climate profiles may support different types of implementation outcomes. Furthermore, these results advance causal theory in implementation science by suggesting that a comprehensive profile is one mechanism through which schools may generate optimal EBP fidelity and sustainment among their staff.

While caution is warranted in interpreting the results of a single study, we note some potential theoretical implications associated with the specific shape of the comprehensive profile. First, the extremely high level of proficient culture suggests that EBP fidelity may be optimized when an organization’s culture engenders a dual focus on improving student well-being and optimizing staff competence. It is important to note that items on the proficiency scale do not refer specifically to EBPs but rather to expectations for teacher competence in effective practices along with a focus on promoting student well-being. This contrasts with other more targeted organizational constructs, such as strategic implementation climate, which assesses the extent to which use of a particular EBP is expected, supported, and rewarded by the organization [74]. Thus, these constructs differ in their specificity and in the extent to which EBP implementation is viewed as a means to an end (i.e., proficiency) versus an end in itself (i.e., implementation climate). It may be that linking EBP implementation to higher-order goals that are valued by providers (e.g., improved client well-being) is helpful (or necessary) to activate the type of practitioner motivation that is necessary to achieve high fidelity to complex EBPs in real-world settings [75]. Conversely, proficient culture may serve as a catalyst for the development of a targeted implementation climate [76]. Second, comprehensive profiles pair a proficient culture with a positive climate, which indicates that staff within these schools perceive that their well-being is supported at work. This pairing may be essential for achieving optimal EBP fidelity because it ensures that providers have the requisite psychological health and well-being necessary to effectively learn and master new practices. Third, the comprehensive profile included average levels of rigidity and resistance as opposed to very low levels of rigidity and resistance. This suggests that some minimum level of rigidity and resistance within a culture may support the achievement of EBP fidelity by providing the requisite structure and healthy skepticism toward competing innovations that are essential to persevering with a new EBP long enough to achieve high fidelity. Additional research is needed to explore these hypotheses and to better understand how the more general organizational characteristics captured by the OSC relate to targeted dimensions of social context such as implementation climate, which are presumed to be most proximal to implementation outcomes.

Third, these results suggest there is not necessarily a linear relationship between organizational culture and climate as has been suggested by some theorists [26, 77]. For example, low levels of rigidity (culture) were associated with both high and low levels of stress (climate), suggesting that the experience of employee autonomy is not universally positive in schools. Similarly, profiles that were high on proficiency (culture) exhibited both high and low levels of stress (climate) depending in part on the accompanying levels of rigidity and resistance (culture). These nuances suggest that while certain dimensions of culture may be more strongly predictive of positive or negative climates in general [37], there are important exceptions in which the level of one dimension of culture may change the effect of another dimension. This is a ripe area for theory development.

Although additional research is needed, results from this study have implications for the implementation of EBPs for youth with autism in elementary schools and the development of strategies to support EBP implementation more broadly. First, these findings suggest that the development of a comprehensive organizational culture and climate profile may serve as a mechanism to support the delivery of EBPs (e.g., DTT and PRT) with high fidelity. Only schools with comprehensive profiles had significantly higher levels of observed fidelity to DTT and PRT and the magnitudes of these relationships were large (*d*’s = .95 to 1.64) suggesting they are practically important. Second, the positive relationship between comprehensive profiles and teacher and staff work attitudes suggests that this type of social context also may support EBP sustainment. Employee retention is a critical factor in maintaining high levels of EBP fidelity [38, 78] and work attitudes are a robust predictor of turnover [39, 40, 79]. To the extent that schools can foster comprehensive culture and climate profiles it is possible that they may be able to support EBP fidelity and sustainment by retaining teachers and staff.

We note that the relationship between OSC profiles and VS implementation was not significant. While there may be many reasons for this, we posit that organizational social context may not be as critical for VS implementation as it is for DTT and PRT. VS implementation is simpler in comparison to DTT and PRT and relies heavily on individual teachers and staff for the preparation of materials (e.g., laminated pictures) and organization (e.g., setting and resetting the schedules), whereas DTT and PRT are more resource intense in terms of staff time (e.g., 10–15 min one-on-one per student) and physical materials (e.g., toys, data sheets), which may require more collaboration and support from other members in the school.

Research in other healthcare settings (e.g., community mental health clinics) indicates that organizational culture and climate are malleable and can be changed with organizational interventions in as little as 18 months [32, 36]. These studies also suggest that improvement in targeted dimensions of organizational culture support improved EBP implementation [31, 34]. However, very little is known about the most efficient or effective ways to change organizational social context or the strategies needed to target specific dimensions. For example, different organizations may need to change their contexts in different ways (e.g., reduce rigidity versus increase proficiency) and consequently, the most effective implementation strategy may vary depending on the organization’s culture and climate profile.

A related area for future research involves identifying predictors of profile membership and understanding the factors that serve as antecedents to the development of comprehensive culture and climate profiles. Results of our post hoc analyses suggest that differences in school size and socioeconomic status of students do not explain why some schools have comprehensive profiles and other schools in the same region have constrained profiles. Identifying factors such as system- and organization-level leadership behaviors and organizational practices that predict profile membership is an important area for future research [4, 80, 81].

### Limitations

It is important to interpret these findings within the context of their limitations. First, we did not conduct a randomized experiment, so the relationships in this study cannot be interpreted causally. Although we established the temporal precedence of organizational culture and climate profiles relative to teacher and staff EBP fidelity, we cannot say that the profiles caused EBP fidelity. It could be that higher EBP fidelity caused different patterns of culture and climate, although this seems unlikely because all teachers and staff received EBP training and support from the same source in a standardized way. More plausibly, a third variable may explain patterns of culture and climate and staff EBP fidelity. For example, the Consolidated Framework for Implementation Research [1] describes several factors (e.g., teacher characteristics, competing initiatives within the school environment) that may confound the relationship between culture and climate profiles and fidelity. This may occur as a confounding effect or as an indirect effect (e.g., leadership causes culture/climate which causes EBP fidelity and staff retention). We attempted to address this in part by controlling for all the policy, resource, and student population differences associated with geographic region in our analysis and by conducting post hoc tests with variables that have theoretical links to culture and climate (i.e., school size and percent of students with free/reduced lunch); however, a more definitive answer awaits experimental research. We are aware of one experiment in behavioral health clinics which showed that planned improvement in a single dimension of culture was associated with increased clinician adoption of EBPs [34]; however, experiments testing the holistic profiles described here have not yet been conducted.

Other limitations include the limited geographic distribution of our sample across northwest and northeast regions in the US. Studies that incorporate nationally representative samples of schools and samples in other countries are needed to replicate and extend our findings. In addition, the use of self-report measures to assess teacher and staff perceptions of organizational culture and climate is sometimes viewed as a weakness; however, we view this as a strength and essential feature of the study because staff respond to their shared perceptions of the social environment rather than the “actual” environment as might be reported by an outside observer [24]. In contrast, fidelity to EBPs is best measured through observational assessments because third-party raters can reliably and validly assess the completion of specified procedures and thereby circumvent self-recall and self-report biases. This study incorporated observer-rated fidelity to three different EBPs and linked these to staffs’ prior, shared perceptions of the social context, providing strong evidence for a link between culture and climate profiles and EBP fidelity. Finally, this study would have been strengthened with qualitative data and member checking around the findings. This is an important area that warrants additional research.

## Conclusion

Results of this study highlight the importance of an organization’s overarching social context and the complex interplay among individual dimensions of organizational culture and climate as they relate to observed fidelity to EBPs and staff work attitudes that support EBP sustainment. The study identified four unique culture and climate profiles that characterized schools (indifferent, constrained, supportive, and comprehensive) and confirmed EBP fidelity was significantly superior in 9% of schools that exhibited a comprehensive profile. Furthermore, teacher and staff work attitudes that support the sustainment of EBPs were also superior in these schools. Examination of the comprehensive profile shape suggests that effective EBP implementation is a function of both a cultural focus on responsiveness to student needs and teacher competence as well as a supportive work environment. These findings highlight important directions for theory development in implementation science and suggest that implementation strategies should cultivate a comprehensive organizational culture and climate profile in order to improve EBP use for youth with autism.

## Additional files

Additional file 1: Standards for Reporting Implementation Studies: the StaRI checklist for completion. (DOCX 78 kb)

Additional file 2: **Table S1.** Raw Correlation Matrix of OSC Dimensions and Fidelity Measures. **Table S2.** OLS Regression Analyses Linking OSC Dimensions to Evidence-Based Practice Fidelity. (DOCX 43 kb)

## Abbreviations

DTT: Discrete trial training
EBP: Evidence-based practice
LPA: Latent profile analysis
OSC: Organizational Social Context
PRT: Pivotal response training
SSA-BIC: Sample size-adjusted Bayesian information criterion
VS: Visual schedules
