# Supplementary material for: Organizational culture and climate profiles: relationships with fidelity to three evidence-based practices for autism in elementary schools
Source: Implement Sci. 2019 Feb 12;14:15. doi: 10.1186/s13012-019-0863-9 (PMC6373074; doi:10.1186/s13012-019-0863-9)
Supplement: Supplementary file 2 — Table S1. Raw Correlation Matrix of OSC Dimensions and Fidelity Measures. Table S2. OLS Regression Analyses Linking OSC Dimensions to Evidence-Based Practice Fidelity. (DOCX 43 kb) [file 13012_2019_863_MOESM2_ESM.docx]

Additional file 2: **Table S1: Raw Correlation Matrix of OSC Dimensions and Fidelity Measures**

| Variable | 1 | 2 | 3 | 4 | 5 | 6 | 7 | 8 |
| --- | --- | --- | --- | --- | --- | --- | --- | --- |
| 1. DTT fidelity |  |  |  |  |  |  |  |  |
| 2. PRT fidelity | .50** |  |  |  |  |  |  |  |
| 3. VS fidelity | .38** | .37** |  |  |  |  |  |  |
| 4. Proficient culture | .10 | .06 | .09 |  |  |  |  |  |
| 5. Rigid culture | -.08 | .03 | -.05 | .29* |  |  |  |  |
| 6. Resistant culture | -.24 | -.02 | -.11 | .07 | .51** |  |  |  |
| 7. Engaged climate | .09 | .07 | .11 | .46** | .01 | -.02 |  |  |
| 8. Functional climate | .17 | .20 | .09 | .46** | -.06 | -.08 | .44** |  |
| 9. Stressful climate | -.13 | -.10 | -.14 | -.13 | .26* | .24 | -.49** | -.50** |

*Note: N* = 62-65 due to some missing values on some OSC subscales. DTT = Discrete Trial Training; OSC = Organizational Social Context; PRT = Pivotal Response Training; VS = Visual Schedules.

***p* < .01

**p* < .05

**Table S2: OLS Regression Analyses Linking OSC Dimensions to Evidence-Based Practice Fidelity**

|  | Outcome | | | | | | | | |
| --- | --- | --- | --- | --- | --- | --- | --- | --- | --- |
|  | DTT Fidelity | | | PRT Fidelity | | | VS Fidelity | | |
| Predictor | *B* | *SE* | *P* | *B* | *SE* | *p* | *B* | *SE* | *p* |
| Proficient culture | .12 | .19 | .536 | .00 | .17 | .995 | .05 | .17 | .785 |
| Rigid culture | -.01 | .19 | .972 | .03 | .17 | .861 | .00 | .17 | .980 |
| Resistant culture | -.25 | .17 | .154 | -.01 | .15 | .967 | -.11 | .15 | .454 |
| Engaged climate | -.03 | .19 | .893 | -.05 | .16 | .771 | .05 | .17 | .763 |
| Functional climate | .15 | .19 | .132 | .24 | .17 | .162 | -.02 | .17 | .894 |
| Stressful climate | -.02 | .19 | .937 | -.02 | .17 | .913 | -.09 | .17 | .620 |
| Model *R^2^* | .09 |  |  | .06 |  |  | .03 |  |  |

*N* = 62. *Note:* DTT = Discrete Trial Training; PRT = Pivotal Response Training; VS = Visual Schedules.
